# Clinical and epidemiologic features of community versus hospital-acquired *Clostridium difficile* infection

**DOI:** 10.1186/1471-2334-14-S7-O25

**Published:** 2014-10-15

**Authors:** Violeta Molagic, Irina Lăpădat, Raluca Mihăilescu, Cristina Popescu, Cătălin Tilişcan, Raluca Jipa, Mihaela Rădulescu, Daniela Munteanu, Adriana Hristea, Ruxandra Moroti, Anca-Ruxandra Negru, Iulia Niculescu, Roxana Petre, Raluca Năstase, Angelica Teniță, Victoria Aramă

**Affiliations:** 1 National Institute for Infectious Diseases "Prof. Dr. Matei Balş", Bucharest, Romania; 2 Carol Davila University of Medicine and Pharmacy, Bucharest, Romania

## Background

*Clostridium difficile* infection (CDI) is an increasingly common hospital-associated infection. There is an increasing awareness in recent years about the impact of community-acquired *Clostridium difficile* infection (CA-CDI).

## Methods

We enrolled all CDI patients admitted to the Adults III department of the National Institute for Infectious Diseases "Prof. Dr. Matei Balş", Bucharest, between January – July 2014. Stool culture, toxin EIA and Cepheid Gene Xpert *C. difficile* test were used for CDI diagnosis. The subjects were divided into two groups: CA-CDI patients (Group 1) and HA-CDI patients (Group 2). Our objective was to describe the clinical, epidemiologic features and outcome of CA-CDI compared to hospital-associated CDIs (HA-CDI) including the ATLAS bedside severity scoring system. Statistical analyses were performed using SPSS Statistics package v.17.

## Results

We included 57 patients with median age 69 years (IQR =54;78). Male/female ratio was 0.72. Most patients (75.4%) presented with an initial CDI episode, the rest having the first (17.5%) or next (2-5) (7%) recurrences. The median value of ATLAS score was 3 (IQR=2;4). Most patients (87.7%) had previously received antibiotic therapy. In 15.8% cases cancer had been previously diagnosed and 17.5% of patients had had recent surgery. *Clostridium difficile* 027 strain was identified in almost all patients.

The patients were treated with vancomycin (73.7%), metronidazole (12.3%), vancomycin/metronidazole association (10.3%); 3.5% received tigecycline. Nine patients (15.8%) were included in the CA-CDI and forty-eight patients (84.2%) had HA-CDI. The number of CA-CDI in the first 7 months of 2014 was about two times higher than in 2013. Group 1 had fewer comorbidities, were younger (median 52 years (IQR 34.5;77) vs. 69.50 years (IQR 55.25;78), p=0.164, had more mild CDI episode (55.6% vs. 33.3%, p=0.26), all had received antibiotics and two cases received proton pomp inhibitors.

Group 1 received more aminopenicillins (33.3%) and less CEPH (11.1%) compared to Group 2 (2.1% and 18.8%, respectively, p=0.06 and p=1). FQ use was similar: 22.2% in Group 1 vs. 18.8% in Group 2. There was one death in HA-CDI. There were no statistical differences between the two groups regarding: sex distribution, median ATLAS score – 3, rates of complicated/recurrent CDI and use of vancomycin or metronidazole treatment.

## Conclusion

Approximately one sixth of CDIs were CA-CDI. These patients were younger, had predominant mild CDI and received more frequently aminopenicillins than those with HA-CDI. We found no significant differences between the two groups regarding *Clostridium difficile* 027 strain prevalence and infection severity.

